# Effect of Loading Strategy on Methylene Blue Encapsulation in Ethosomes: A Comparative Study of Aqueous and Ethanol Phases

**DOI:** 10.3390/mps9020057

**Published:** 2026-04-02

**Authors:** Dmitry Yakovlev, Kanamat Efendiev, Polina Alekseeva, Vladimir Makarov, Kirill Linkov, Anna Lukianova, Vladimir Oleinikov, Victor Loschenov

**Affiliations:** 1 Prokhorov General Physics Institute, Russian Academy of Science, 119991 Moscow, Russia; kanamatius@mail.ru (K.E.); alekseeva.polina2012@mail.ru (P.A.); vi.makarov@physics.msu.ru (V.M.); loschenov@mail.ru (V.L.); 2 Shemyakin-Ovchinnikov Institute of Bioorganic Chemistry, Russian Academy of Science, 117997 Moscow, Russia; voleinik@mail.ru; 3 Department of Laser Micro-, Nano-, and Biotechnology, Institute of Engineering Physics for Biomedicine, National Research Nuclear University “MEPhI”, 115409 Moscow, Russia; anneta_loginova@bk.ru; 4 Institute of Mathematics and Natural Sciences, Kabardino-Balkarian State University, 360004 Nalchik, Russia; 5 Laboratory of Neurobiology and Tissue Engineering, Brain Science Institute, Research Center of Neurology, 125367 Moscow, Russia

**Keywords:** ethosomes, methylene blue, loading strategy, encapsulation efficiency, vesicular drug delivery, photodynamic therapy, transdermal delivery, colloidal stability

## Abstract

This study presents a comparative analysis of the effect of methylene blue (MB) loading strategy on the physicochemical and colloidal properties of ethosomes prepared by the cold method. Two synthesis protocols differing in the phase of introduction of the cationic hydrophilic dye were investigated: a classical approach with MB loading into the aqueous phase and an alternative approach involving MB incorporation into the ethanolic lipid phase. It is shown that the loading strategy is a critical technological factor determining vesicle size, encapsulation efficiency, loading capacity, and electrokinetic properties of the systems. The alternative method results in the formation of smaller ethosomes (R_h_ ≈ 78 nm) compared to the classical protocol (R_h_ ≈ 96 nm), but is accompanied by a lower encapsulation efficiency (EE ≈ 36% versus 48%). The results indicate that a reduction in vesicle size does not necessarily lead to higher encapsulation of hydrophilic cationic MB and may be associated with a decrease in the total internal aqueous volume as well as an increased contribution of a weakly bound surface-associated dye fraction. Spectral analysis indicates the preservation of a predominantly monomeric form of MB within ethosomes, while differences in ζ-potential suggest distinct localization of the dye within the vesicular systems. Overall, the results highlight the importance of optimizing the loading protocol in the development of ethosomal drug delivery systems for photodynamic therapy and topical applications.

## 1. Introduction

The development of effective local (topical, transdermal, and intradermal) drug delivery systems for hydrophilic and cationic low-molecular-weight compounds remains one of the most challenging tasks in modern pharmaceutical technology [1,2]. The principal limiting factor is the stratum corneum, which exhibits a pronounced lipophilic barrier function and significantly restricts the penetration of polar molecules in the absence of specialized carriers or penetration-enhancing approaches [1,2,3]. From a clinical and applied perspective, this results in low and poorly reproducible accumulation of active substances in target tissues, the need to increase local doses or application frequency, and a strong dependence of therapeutic efficacy on the administration protocol [1,3]. Various physical and pharmaceutical strategies have been proposed to overcome the skin barrier; however, each of them is associated with limitations related to safety, usability, and formulation stability [3].

An additional systemic challenge is the low encapsulation efficiency of small hydrophilic molecules in vesicular systems. During passive formation of lipid vesicles, a substantial fraction of the active compound remains in the external phase or is rapidly released, leading to low payload and reduced formulation stability [4]. Therefore, the choice of loading strategy and carrier composition represents a critically important stage of formulation development [5]. In the context of photodynamic therapy (PDT), the selection of a photosensitizer (PS) is a key aspect of drug formulation, since the photophysical properties of the molecule, its redox behavior, charge, and aggregation tendency directly determine the requirements for the carrier system and encapsulation technology [6,7]. In the present study, methylene blue (MB) was chosen as a model PS due to its clinical relevance, practical availability, and pronounced photodynamic activity [8,9]. MB is widely used in PDT and antimicrobial photodynamic inactivation (aPDI). Its main advantages include absorption in the red spectral region, efficient generation of reactive oxygen species (ROS) upon light irradiation, and relatively low dark toxicity [8,9]. At the same time, the efficacy of MB critically depends on its microenvironment and concentration-dependent effects [6]. In aqueous and polar media, MB is prone to self-aggregation, which is accompanied by changes in absorption spectra and a decrease in the quantum efficiency of singlet oxygen generation [10,11].

The photoactivity of MB is also sensitive to redox conditions and electrostatic interactions, affecting its distribution between carrier compartments and the availability of the photoactive fraction [12,13]. Our previous studies have confirmed the high sensitivity of the photophysical properties and biological effects of MB to its microenvironment and demonstrated that optimization of the drug formulation can significantly alter both the photophysics and biological activity of the PS [8,14,15,16,17,18]. Various nanoplatforms, including liposomes, transfersomes, ethosomes, and niosomes, have been employed for the delivery of hydrophilic and cationic small molecules into the skin [19,20,21,22,23]. Among them, ethosomes are of particular interest due to their ability to combine enhanced transdermal penetration with relatively mild encapsulation conditions [24,25,26,27,28,29]. The size characteristics of ethosomes and their encapsulation efficiency strongly depend on system composition and preparation method [26,27,28,29]. Previously, we demonstrated the feasibility of producing MB-loaded ethosomes and their improved skin penetration compared to non-carrier systems [18]. The loading strategy of the active compound during vesicle formation is a key factor governing molecular distribution between system compartments and the reproducibility of loading parameters [30,31,32,33,34,35]. The functional state of MB as a PS in biologically relevant environments is of fundamental importance for PDT, as it directly correlates with ROS generation and the reproducibility of the therapeutic effect [36,37,38]. In practical applications, the effectiveness of MB-mediated PDT in skin treatments is often limited by insufficient MB retention, aggregation, and reduction to the leuco-form [12,39,40].

Thus, optimization of the preparation protocol for MB-loaded ethosomes, with explicit consideration of the loading strategy, represents a rational approach to improving the stability of the photoactive monomeric fraction and the reproducibility of topical PDT.

Therefore, the aim of the present study was to compare two loading strategies for incorporating methylene blue into ethosomes prepared by the cold method and to evaluate their influence on vesicle size, encapsulation efficiency, loading capacity, and electrokinetic properties.

## 2. Materials and Methods

Lecithin (Serva, Heidelberg, Germany), polysorbate 80 (Tween 80, reagent grade, Khimprom-M, Moscow, Russia), methylene blue (MB; Samaramedpromtorg, Samara, Russia), and ethanol (96%) were used as received without additional purification. A stock solution of MB was prepared in distilled water at a concentration of 10 g/L (1%) and used immediately after preparation, without a storage step. The use of a freshly prepared solution ensured preservation of its physicochemical properties and homogeneity, which was critical for obtaining reproducible results in ethosome synthesis. In all experiments, 100 μL of the MB stock solution was added to the system, corresponding to a nominal amount of 1 mg of the dye introduced.

### 2.1. Synthesis of MB-Loaded Ethosomes

**Figure 1:**
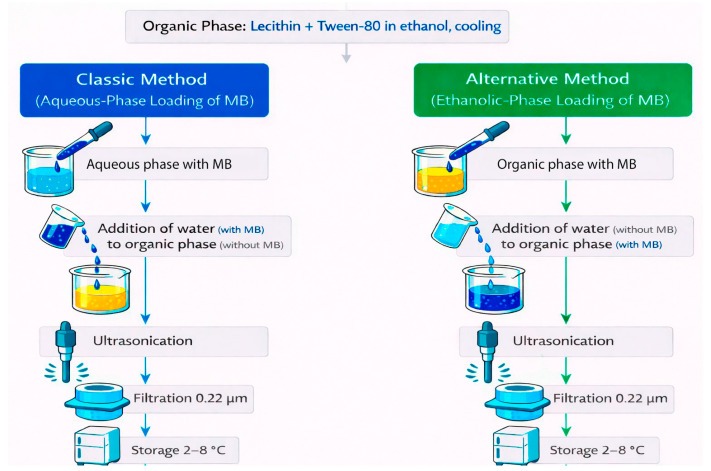
Schematic representation of two ethosomes synthesis approaches.

Ethosomes were prepared using a modified cold method in two variants differing in the phase of methylene blue (MB) introduction: the classical method (aqueous-phase loading) and the alternative method (organic-phase/ethanolic loading). A schematic representation of both approaches is shown in Figure 1.

The scheme shown in Figure 1 illustrates the sequence of stages in MB-ethosome formation and highlights the fundamental difference between the classical and alternative methods, which lies in the phase (aqueous vs. ethanolic) of introducing MB into the system.

*General Procedure for Organic Phase Preparation*

In all experimental variants, the organic phase was prepared by dissolving 100 mg of lecithin and 10 µL of Tween-80 in 9 mL of ethanol at 40 °C under continuous stirring until complete dissolution of the lipid component and formation of a homogeneous solution. The solution was then cooled to 4 °C. After the introduction of the aqueous phase, the total system volume was approximately 20 mL. For the calculation of the loading capacity (LC), the mass of the lipid material (W) was taken as 100 mg.

*Classical Method (Aqueous Phase Loading)*

To prepare the aqueous phase, 100 µL of the MB stock solution (1%) was added to 11 mL of distilled water, corresponding to a nominal initial dye amount of A_0_ = 1 mg (the initial amount of the compound introduced into the system prior to encapsulation). The organic phase, cooled to 8–10 °C, was stirred using a magnetic stirrer (BioSan, Riga, Latvia) at 700 rpm. The aqueous phase (11 mL) was added dropwise to the organic phase over 10–15 min under continuous, vigorous stirring. The resulting ethosome suspension was subjected to ultrasonic treatment in a pulsed mode (5 s on/5 s off) for 15–20 min using an IKA Ultra-turrax T-18 homogenizer (IKA, Staufen im Breisgau, Germany). The system temperature was maintained at or below 10 °C. After sonication, the suspension was filtered through a membrane filter with a pore size of 0.22 µm (Millipore, Burlington, VT, USA). The obtained samples were stored in a dark place at 5–8 °C.

*Alternative Method (Organic Phase/Ethanol Loading)*

**Figure 2:**
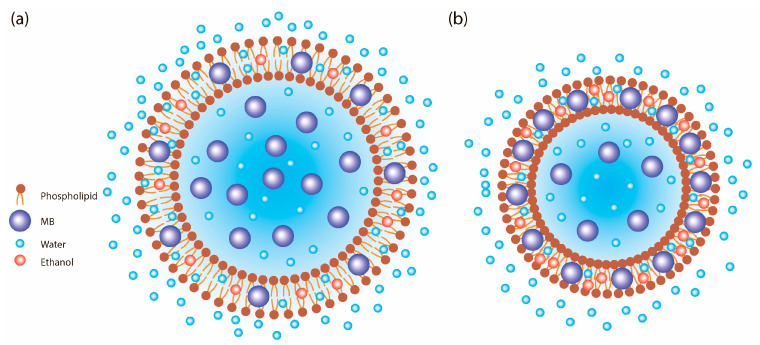
(**a**) Schematic illustration of an MB ethosome (with phospholipid, MB, ethanol, and water) prepared by the classical synthesis method, (**b**) Schematic illustration of an ethosome prepared by the alternative synthesis method.

In the alternative protocol, MB was pre-introduced into the organic phase. To the cooled (4 °C) ethanolic solution of lecithin and Tween-80, 100 µL of the MB stock solution (1%) was added, corresponding to a nominal initial dye amount of A_0_ = 1 mg. Separately, 11 mL of distilled water was prepared. The aqueous phase was added dropwise to the organic phase under vigorous stirring at a temperature of 8–10 °C over a period of 10–15 min (Figure 2). The subsequent ultrasonic treatment and storage were performed according to the procedure described for the classical method.

### 2.2. Particle Size Determination

The hydrodynamic radius (Rh) of the ethosomes was determined by dynamic light scattering (DLS) using a “Photocor Complex” instrument (Photocor, Moscow, Russia) at a temperature of 25 °C. Prior to measurements, samples were diluted with distilled water to an optically acceptable concentration when necessary. Each measurement was performed in triplicate. The results are presented as the mean R_h_ ± standard deviation, while the polydispersity index (PDI) was used to characterize the width of the particle size distribution. Measurements were taken immediately after suspension preparation, as well as after storage at 2–8 °C for 1 and 2 weeks to assess the colloidal stability of the systems.

### 2.3. Spectral and Fluorescence Analysis

The optical density (OD) of MB in filtrates and solutions was recorded using a Hitachi U-3410 spectrophotometer (Hitachi, Tokyo, Japan). Quantitative determination of MB concentration was performed using a calibration curve constructed over the relevant wavelength range, including the analytical wavelength of 665 nm. Samples were diluted as necessary to fall within the linear range of the calibration plot, after which the MB concentration (C, mg/L) was calculated.

Photoluminescence (PL) spectra of MB-loaded ethosomes were recorded using a Shimadzu RF-5301PC spectrophotometer (Shimadzu, Kyoto, Japan) with identical acquisition parameters for all samples.

### 2.4. Determination of Non-Encapsulated MB Amount

The amount of non-encapsulated (free) MB was assessed using a sequential ultrafiltration method. A 0.4 mL aliquot was taken from the initial ethosome suspension and transferred into a 0.5 mL Amicon Ultra centrifugal filter (MilliporeSigma, Burlington, MA, USA) unit with a molecular weight cutoff of 100 kDa.

1. The first centrifugation was performed at 10,000 rpm for 20 min, after which the filtrate was collected for subsequent analysis.
2. The retentate was reconstituted to the initial volume of 0.4 mL using distilled water, and a second centrifugation was carried out under the same conditions, yielding a second filtrate.

The concentration of MB in each fraction, C_i_ (mg/L), was determined spectrophotometrically using the calibration curve. The amount of non-encapsulated MB in each fraction was calculated using the formula:

A_u,i_ = C_i_⋅V_i_, (1)

where C_i_ is the concentration of the corresponding filtrate, and V_i_ is the volume of the corresponding filtrate.

The total amount of non-encapsulated MB in the analyzed 0.4 mL sample was calculated as:

(2) $$A_{u}\;=\;\sum_{i}A_{u,i}$$

### 2.5. Calculation of Encapsulation Efficiency and Loading Capacity

The amount of encapsulated MB was calculated using the formula:

A_e_ = A_0_ − A_u_ (3)

where A_0_ = 1 mg is the nominal amount of MB introduced, and A_u_ is the experimentally determined amount of non-encapsulated dye.

The encapsulation efficiency (EE) and loading capacity (LC) of MB in ethosomes were calculated using the following formulas, employing only the nominal amount of dye introduced, A_0_:

EE (%) = A_e_/A_0_ × 100, (4)

LC (%) = A_e_/W × 100 (5)

where W = 100 mg is the mass of lecithin.

For both the aqueous-phase and organic-phase loading methods, the EE and LC values were calculated separately according to the scheme described above.

### 2.6. Statistical Data Analysis

The goodness-of-fit of the experimental particle size distributions was evaluated using the Pearson’s χ^2^ criterion:

(6) $$\chi^{2}=\sum_{i}\frac{{\left(I_{i}^{exp}-\;I_{i}^{fit}\right)}^{2}}{max(I_{i}^{fit},\;ℇ)}$$

where $I_{i}^{exp}$ and $I_{i}^{fit}$ are the normalized experimental and fitted intensity values, respectively, and $ℇ=10^{-6}$ is introduced to prevent division by zero.

### 2.7. Determination of ζ-Potential of Ethosomes

The ζ-potential of the ethosomes was determined by electrophoretic light scattering using a Zetasizer analyzer (Malvern Panalytical, Malvern, UK). Measurements were performed at 25 °C using disposable ζ-potential cuvettes. For each sample, 12 runs were recorded, and the mean ζ-potential value was calculated.

## 3. Results and Discussion

### 3.1. Spectral Properties of MB in Ethosomes

**Figure 3:**
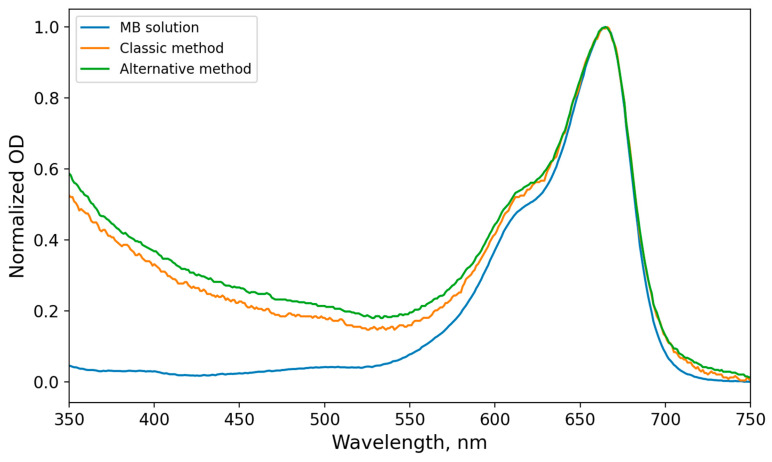
Optical density (OD) spectra of free MB and MB encapsulated in ethosomes, normalized to the absorption maximum.

To evaluate the effect of MB incorporation into ethosomes on its photophysical spectral characteristics, absorption and photoluminescence spectra of free MB and MB encapsulated in ethosomes were recorded. A comparison of the normalized absorption spectra of MB in solution and in ethosomes prepared by the classical aqueous-phase loading method and the alternative organic-phase (ethanolic) loading method demonstrated a high degree of similarity (Figure 3). This indicates the absence of pronounced changes in the electronic transitions of the MB molecule upon incorporation into ethosomes and confirms the preservation of the predominantly monomeric form of the dye in the ground state, with no detectable aggregation within the sensitivity limits of the applied method. The noticeable changes in the optical density spectra in the hypsochromic region are associated with the presence of organic components introduced during ethosome synthesis.

**Figure 4:**
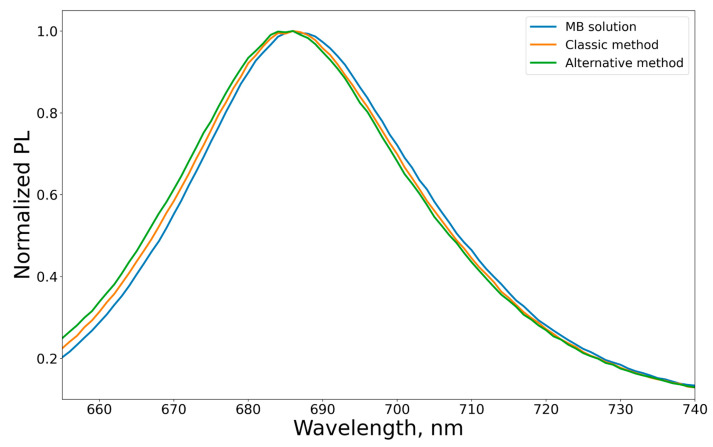
Photoluminescence spectra of free MB and MB encapsulated in ethosomes, normalized to the emission maximum (λ_ex_ = 635 nm).

In Figure 3, all spectra were recorded under identical experimental conditions and normalized to the absorption maximum at 665 nm, which allows a correct comparison of the shape and position of the spectral bands independently of the absolute MB concentration in the samples. At the same time, the photoluminescence (PL) spectra exhibit a slight hypsochromic shift (≈2 nm) of the emission maximum for MB encapsulated in ethosomes compared to free MB (Figure 4).

Such changes may be attributed to several factors:

1. Incorporation of MB into the vesicular system alters its microenvironment and interfacial distribution upon interaction with the vesicle membrane, which can modify the properties of the excited state and the quenching pathways;
2. Contributions from the secondary inner filter effect (self-absorption) in dispersed systems may lead to concentration-dependent shifts in the observed emission maximum toward the red spectral region [13].

### 3.2. Size Characteristics of Ethosomes

**Figure 5:**
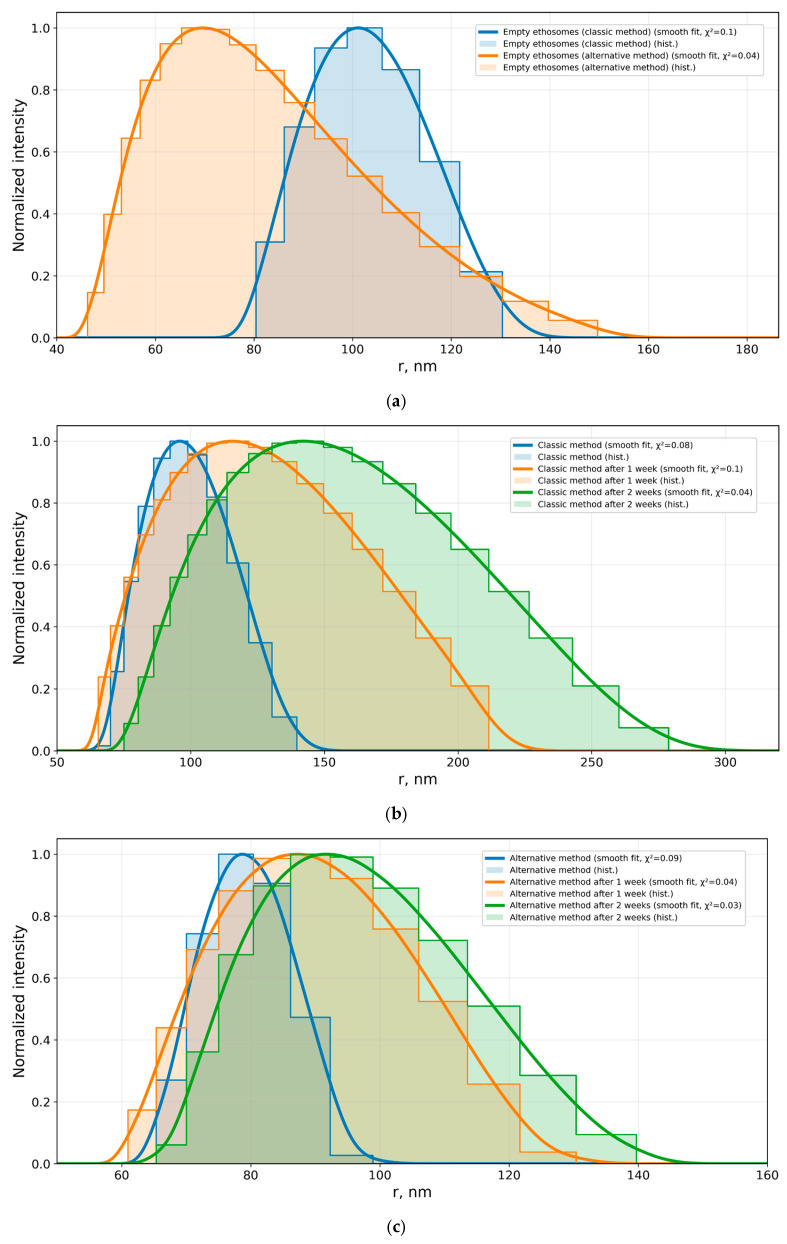
Size distributions of ethosomes: (**a**) empty ethosomes; (**b**) MB-loaded ethosomes prepared by the classical synthesis method; (**c**) MB-loaded ethosomes prepared by the alternative synthesis method.

Within the study, size distributions were obtained for empty ethosomes as well as for MB-loaded ethosomes prepared using the classical and alternative methods (Figure 5).

It is worth noting that, in terms of size distribution, empty ethosomes were closer to those obtained by the alternative method (Figure 5a). This can be interpreted as a structural organization more similar to the “base” lipid–ethanol–water system, whereas in the classical method, the introduction of MB via the aqueous phase may increase the likelihood of forming a small fraction of unstable, larger structures during storage. This behavior may be associated with changes in interfacial interactions and self-assembly kinetics, which are reflected in DLS measurements as an enhancement of the right-hand tail of the size distribution. The overall influence of composition and preparation technology on D_h_, PDI, and ethosome stability is also emphasized in the review literature [41].

The alternative method (introduction of MB into the ethanolic lipid phase) resulted in the formation of smaller vesicles with a hydrodynamic radius of R_h_ = 78 ± 5 nm (Figure 5c), whereas larger particles were observed for the classical method (introduction of MB via the aqueous phase), with R_h_ = 96 ± 9 nm (Figure 5b).

The agreement between the experimental DLS histograms and the corresponding smoothed size distributions was evaluated using the Pearson χ^2^ criterion (Equation (6)). In all cases, the obtained χ^2^ values were below 1 (χ^2^ ≲ 1), indicating good agreement between the approximations and the experimental data and confirming the adequacy of the applied smoothing procedure for describing the particle size distribution profiles.

The differences in the hydrodynamic radius of MB-loaded ethosomes obtained using the classical and alternative protocols can be explained by the fact that ethosome size is sensitive not only to composition (phospholipid/ethanol/surfactant), but also to the preparation procedure, including the order of phase mixing and self-assembly conditions. Reviews on ethosomes emphasize that the preparation technique and technological parameters (mode of aqueous phase addition, mixing conditions, and subsequent filtration) have a significant impact on the size and polydispersity index of the final dispersion [41].

The observed differences in vesicle size may also be attributed to the specific molecular interactions between MB and the lipid component at different stages of synthesis. In the alternative method, the cationic dye comes into contact with phospholipids already at the phase-mixing stage, prior to the final vesicle self-assembly. In contrast, in the classical protocol, MB is introduced into the system as part of the aqueous phase, implying a different mode of distribution. The different order of introduction of the cationic agent can modulate the kinetics of ethosome formation and alter membrane curvature, thereby determining the final average hydrodynamic radius. The influence of technological parameters and ethanol concentration on the structural characteristics of ethosomes is widely discussed in the literature; in particular, variations in synthesis conditions have been shown to be a determining factor in the regulation of vesicle size [42].

A number of studies report an inverse relationship between ethanol concentration and ethosome size [13,43,44,45,46,47,48,49,50]. In particular, Bendas and Tadros demonstrated that the average vesicle diameter in an ethosomal formulation containing 40% ethanol was 44.6% smaller than that of classical liposomes [51]. However, exceeding the optimal ethanol concentration leads to bilayer destabilization, which is accompanied by a slight increase in vesicle size and a sharp decrease in encapsulation efficiency; further increases in ethanol content result in complete vesicle dissolution.

The mechanism underlying particle size reduction is commonly attributed to two main factors. First, high ethanol concentrations induce interdigitation of lipid hydrocarbon chains, thereby reducing membrane thickness and the overall vesicle volume. Second, the presence of ethanol affects the surface charge of the system, providing electrostatic and steric stabilization that inhibits aggregation and promotes the formation of smaller particles [52,53,54,55].

### 3.3. Encapsulation Efficiency and Loading Capacity

Ethosomes were prepared using two variants of the cold method under identical composition and process conditions; the only difference was the photosensitizer (PS) loading strategy: in the classical approach, MB was introduced into the aqueous phase, whereas in the alternative approach, MB was introduced into the organic (ethanolic) phase followed by dropwise addition of water.

According to DLS data, the alternative method produced smaller vesicles (R_h_ = 78 ± 5 nm) compared to the classical method (R_h_ = 96 ± 9 nm). After standardized removal of free dye by sequential ultrafiltration, the classical protocol exhibited higher loading parameters than the alternative protocol (Table 1).

The lower loading observed at smaller Rh in the alternative method may be explained by the fact that, for the hydrophilic cationic MB, a reduction in vesicle size is not equivalent to an increase in encapsulation degree. At a fixed lipid mass, a decrease in Rh may be accompanied by a reduction in the total internal aqueous volume of the vesicles and/or by redistribution of MB into a near-surface, weakly bound fraction, which is subsequently removed during ultrafiltration and accounted for as free dye.

In the literature, reported encapsulation efficiencies for ethosomes vary over a wide range and depend on the nature of the active compound, system composition, and the method used to separate the free fraction. In some studies, EE values for ethosomal systems range from approximately ~36% to ~96% depending on formulation parameters (phospholipid and ethanol concentrations) and preparation conditions [39]. For optimized ethosomal formulations (e.g., domperidone), EE values of around ~70% have been reported, with EE varying between different formulations [12]. Comparative studies on ultradeformable vesicles have also shown that the type of system affects EE, with higher values reported for transethosomes than for ethosomes (approximately ~84% versus ~72%) [51]. Against this background, the more moderate values obtained in the present study (EE ~36–48% after ultrafiltration) can be interpreted as a “conservative” estimate, since for hydrophilic cationic MB a substantial fraction of the dye may reside in a weakly bound, near-surface fraction and be removed during strict separation of free dye. In addition, differences between studies are amplified by the use of different EE determination methods (ultracentrifugation, dialysis, ultrafiltration, etc.) [49].

### 3.4. ζ-Potential of Ethosomes

For MB-loaded ethosomes obtained using both protocols, a negative ζ-potential was observed (Table 2), which is characteristic of vesicles based on phosphatidylcholine and ethanol. In such systems, the negative surface charge arises from the orientation of polar lipid headgroups and the presence of ethanol, which contributes to surface stabilization [24]. Empty phosphatidylcholine-based ethosomes are also typically characterized by a negative ζ-potential, which is attributed to the orientation of phospholipid headgroups and the influence of ethanol on membrane organization and surface charge, as widely reported in the literature [24,41,50,51].

The observed shift in the ζ-potential toward less negative values in the alternative method may reflect differences in the surface architecture of the nanosystem. Considering the cationic nature of the MB^+^ molecule, its presence can lead to partial electrostatic shielding of negatively charged phosphate groups at the interface [24]. The “weaker” negative potential observed for the alternative method may indicate a larger fraction of MB associated with the outer surface of the vesicle membrane. In contrast, the more negative ζ-potential measured for the classical method is consistent with preferential localization of MB within the internal aqueous compartment of the vesicles, which minimizes its influence on the charge of the external surface.

Free methylene blue in aqueous solution exhibits a positive ζ-potential as a cationic dye, and its incorporation into negatively charged ethosomes radically alters the electrokinetic profile of the system [24]. Such “masking” of the positive charge of MB within a negatively charged nanoparticle represents an advantage, as it reduces nonspecific binding to plasma proteins while simultaneously enhancing interactions with specific biological substrates, such as components of biofilm matrices or cell membranes, whose interactions are governed by electrostatic forces [54].

Although the present results provide indirect evidence of different MB localization depending on the loading strategy, direct confirmation of dye distribution within the vesicles would require additional experimental approaches. Techniques such as fluorescence quenching with membrane-impermeable quenchers, fluorescence lifetime spectroscopy, Förster resonance energy transfer (FRET), or confocal fluorescence microscopy may be useful for distinguishing between surface-associated and internally localized dye fractions [13,52,54,55].

### 3.5. Colloidal Stability During Storage

Analysis of the size distributions (Table 3) after storage at 4 °C showed that changes in the mean hydrodynamic size do not necessarily indicate that the system “degrades” via a coarse aggregation pathway. The samples were stored at 4 °C in the dark to minimize possible photodegradation of methylene blue.

This behavior is expected for DLS measurements, since the method is inherently based on intensity-weighted size distributions, and the contribution of larger objects to light scattering increases disproportionately. As a result, even a small fraction of larger particles or vesicles can significantly shift the mean size value and “stretch” the right-hand tail of the distribution while the position of the main peak remains relatively stable. This limitation of DLS—namely, its high sensitivity to minor aggregate populations and the influence of the averaging parameter selection (e.g., Z-average)—is discussed in detail in methodological sources and DLS guidelines.

In the experimental study, storage at 4 °C led to an enhancement of the right-hand tail of the size distribution, which was particularly pronounced for ethosomes prepared by the classical method (Figure 5b). In contrast, for the alternative method, the main population remained within the nanoscale range, and the contribution of the larger fraction was less pronounced (Figure 5c). This pattern of changes is consistent with literature data on the physical stability of phospholipid vesicles, where moderate structural rearrangements during storage—including partial fusion and coalescence of individual vesicles—are possible. Such processes manifest as growth of the distribution “tail” and a gradual drift in size, without a mandatory multiplicative increase in the entire population [56].

Numerous studies confirm the high stability of ethosomal systems both as original suspensions and as components of various dosage forms, including gels, patches, and creams [56,57,58,59,60,61]. Although only a limited number of reports cover storage periods exceeding one year, the available data indicate the long-term stability of vesicular structures. For example, classical ethosomal suspensions of minoxidil and testosterone [43], as well as trihexyphenidyl hydrochloride [62], retained stability for up to two years. Similarly, an erythromycin-loaded ethosomal gel remained stable for approximately one year [41]. Nevertheless, further systematic studies are required to comprehensively assess the long-term stability of ethosomes, particularly within complex composite formulations.

## 4. Conclusions

This study demonstrates that the strategy of methylene blue (MB) introduction during the cold preparation of ethosomes is a key technological factor determining the overall set of colloidal and physicochemical characteristics of the resulting systems. It was established that the alternative protocol, involving the introduction of MB into the ethanolic lipid phase followed by dropwise addition of the aqueous phase, leads to the formation of ethosomes with a smaller hydrodynamic radius (R_h_ = 77 ± 5 nm), compared to the classical method, in which the dye is loaded via the aqueous phase (R_h_ = 100 ± 9 nm).

Under standardized and methodologically rigorous separation of free dye by sequential ultrafiltration, the classical protocol exhibited higher loading parameters (EE = 48%; LC = 0.48%) than the alternative one (EE = 36%; LC = 0.36%). The lower MB loading observed at smaller Rh in the alternative method may be attributed to a reduction in the total internal aqueous volume of the vesicles and/or an increased contribution of weakly bound, surface-associated MB that is removed during ultrafiltration.

In systems obtained by both the classical and alternative methods, a negative ζ-potential characteristic of phospholipid–ethanol-based ethosomes was preserved; however, its magnitude strongly depended on the MB loading strategy (alternative method: −23 ± 4 mV; classical method: −34 ± 5 mV). The more negative ζ-potential values observed for MB-loaded ethosomes prepared by the classical method may indicate preferential localization of the dye within the internal aqueous compartment of the vesicles, whereas the shift toward less negative ζ-potential values in the alternative approach suggests a more pronounced surface association of the cationic dye. These differences may potentially influence the colloidal stability of the systems and the nature of their electrostatic interactions with biological substrates.

Spectral analysis showed that incorporation of MB into ethosomes does not lead to significant changes in its absorption spectra, indicating preservation of the electronic transitions of the dye in the ground state. At the same time, minor differences observed in the luminescence spectra point to the influence of the dye microenvironment and/or inner filter optical effects characteristic of dispersed colloidal systems.

Taken together, the results demonstrate that the alternative method enables a reduction in the size of MB-loaded ethosomes compared to the classical synthesis protocol, which is of fundamental importance for the development of ethosomal carriers for photodynamic therapy and other biomedical applications.

## Figures and Tables

**Table 1:** Encapsulation Efficiency of MB and Loading Capacity of Ethosomes.

	Classical Method	Alternative Method
EE, %	48 ± 4	36 ± 4
LC, %	0.48 ± 0.04	0.36 ± 0.04

Abbreviations: MB—methylene blue; EE—encapsulation efficiency; LC—loading capacity.

**Table 2:** ζ-Potential of ethosomes prepared by two different methods.

	Classical Method	Alternative Method
ζ-Potential, mV	−34 ± 5	−23 ± 4

**Table 3:** Size distribution of ethosomes over time.

Type of Ethosomes	Classical Method (R_h_, nm)	PDI (Classical Method)	Alternative Method (R_h_, nm)	PDI (Alternative Method)
Empty ethosomes	102 ± 8	0.14 ± 0.02	73 ± 7	0.12 ± 0.02
Ethosomes with MB (immediately after synthesis)	96 ± 9	0.16 ± 0.03	78 ± 5	0.11 ± 0.02
Ethosomes with MB (after 1 week)	117 ± 7	0.18 ± 0.03	89 ± 5	0.13 ± 0.02
Ethosomes with MB (after 2 weeks)	144 ± 10	0.22 ± 0.04	95 ± 6	0.15 ± 0.03

## Data Availability

The data presented in this study are available on request from the corresponding author.

## Abbreviations

The following abbreviations are used in this manuscript:

MB	Methylene blue
PS	Photosensitizer
PDT	Photodynamic therapy
aPDI	Antimicrobial photodynamic inactivation
ROS	Reactive oxygen species
SC	Stratum corneum
EE	Encapsulation efficiency
LC	Loading capacity
Rh	Hydrodynamic radius
Dh	Hydrodynamic diameter
PDI	Polydispersity index
DLS	Dynamic light scattering
OD	Optical density
PL	Photoluminescence
A_0_	Nominal amount of dye introduced
A_e_	Amount of encapsulated dye
A_u_	Amount of unencapsulated dye
A_u,i_	Amount of unencapsulated dye in fraction *i*
C_i_	Concentration of dye in fraction *i*
V_i_	Volume of fraction *i*
W	Lipid mass
χ^2^	Pearson chi-squared criterion
λ_ex_	Excitation wavelength
kDa	Kilodalton
